# M. Vautier’s Case of Recovery from Poisoning with Corrosive Sublimate

**Published:** 1842-11-19

**Authors:** 


*Case of Recovery from Poisoning with Corrosive Sublimate.* By M. Vautier.—A man aged 68, in a fit of despair, bruised down about a drachm of corrosive sublimate, put it into a ripe pear, and swallowed it. He soon after began to experience a severe smarting in his throat, and a burning sensation in his stomach, followed by vomiting. When first seen about three-quarters of an hour after swallowing the poison, his face was pale, with an expression of anxiety and suffering, his limbs were cold, and continually tossed about, and the pulse was small, but hard and intermitting. The matters which he vomited had a whitish appearance, were tenacious like albumen, and stretched in long strings from his mouth, and were mixed with portions of the pear. Emetics were freely administered, after which he had the white of six eggs, which he also vomited. After this, no other medicine was given; he was simply confined to barley-water, and in three days he recovered. M. Vautier attributes the speedy recovery to the corrosive sublimate having been taken in the solid form, and along with food. As soon as the natural fluids of the stomach had dissolved any portion of the poison, and allowed it to come in contact with the stomach, vomiting was excited, and the greater portion of the poison expelled before it could exert its full power on the system.—*Ibid, from Journ. de Pharm.* April, 1842.

